# Supplementary material for: Identification of photoperiod-induced specific miRNAs in the adrenal glands of Sunite sheep (Ovis aries)
Source: Front Vet Sci. 2022 Jul 22;9:888207. doi: 10.3389/fvets.2022.888207 (PMC9354845; doi:10.3389/fvets.2022.888207)
Supplement: Supplementary file 1 [file Data_Sheet_1.ZIP › Supplementary file/miRDeep2 file.zip!/miRDeep2 file/Know_Structure/LP42A2/oar-mir-376b.pdf]

miRBase precursor : oar-mir-376b
Total read count : 704
oar-miR-376e-5p read count : 280
oar-miR-376b-5p read count : 280
oar-miR-376b-3p read count : 144
remaining reads : 0

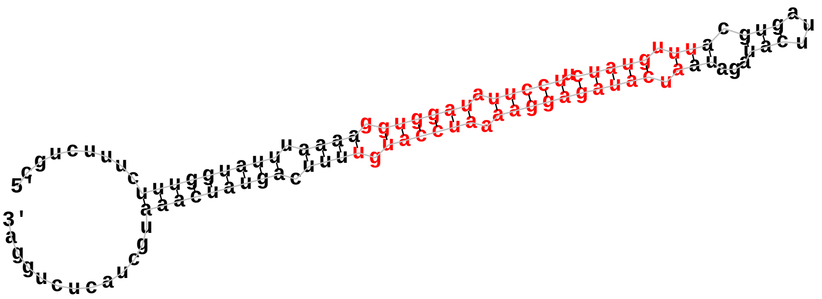

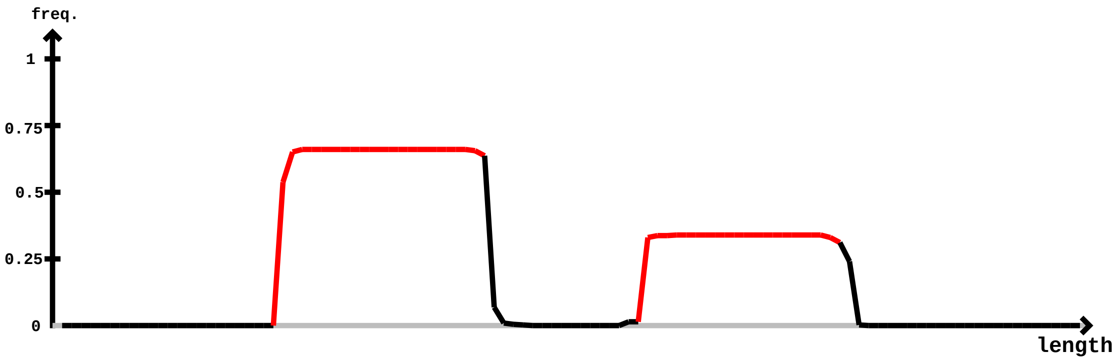


```
                                                             oar-miR-376b-3p
                        oar-miR-376b-5p
                        oar-miR-376e-5p
5'- cgucuuucuuugguauuuaaaagguggauauuccuucuauguuuacgugauucauagauaaucauagaggaaaauccauguuuucaguaucaaaugcuacucugga   -3'   exp
    ........(((((((((.((((.((((((.(((((.(((((.(((.(((...)))...))).)))))))))).)))))).)))).)))))))))............    reads   mm   sample
    ......................gguggauauuccuucuaugu................................................................    2       0    seq
    ......................gguggauauuccuucuauguu...............................................................    7       0    seq
    ......................gguggauauuccuucuauguuu..............................................................    200     0    seq
    ......................gguggauauuccuucuauguuua.............................................................    19      0    seq
    .......................guggauauuccuucuauguu...............................................................    1       0    seq
    .......................guggauauuccuucuauguuu..............................................................    41      0    seq
    .......................guggauauuccuucuauguuua.............................................................    6       0    seq
    ........................uggauauuccuucuauguuuac............................................................    2       0    seq
    ........................uggauauuccuucuauguuuacg...........................................................    1       0    seq
    ........................uggauauuccuucuauguuuacgu..........................................................    1       0    seq
    ..........................................................uaaucauagaggaaaauccau...........................    1       0    seq
    ..........................................................uaaucauagaggaaaauccaug..........................    4       0    seq
    ..........................................................uaaucauagaggaaaauccaugu.........................    1       0    seq
    ............................................................aucauagaggaaaauccau...........................    3       0    seq
    ............................................................aucauagaggaaaauccaug..........................    4       0    seq
    ............................................................aucauagaggaaaauccaugu.........................    29      0    seq
    ............................................................aucauagaggaaaauccauguu........................    98      0    seq
    .............................................................ucauagaggaaaauccauguu........................    2       0    seq
    .............................................................ucauagaggaaaauccauguuu.......................    1       0    seq
    ...............................................................auagaggaaaauccauguu........................    1       0    seq
```
